# Disentangling the relationship of gut microbiota, functional gastrointestinal disorders and autism: a case–control study on prepubertal Chinese boys

**DOI:** 10.1038/s41598-022-14785-8

**Published:** 2022-06-23

**Authors:** Oscar W. H. Wong, Angela M. W. Lam, Brian P. N. Or, Flora Y. M. Mo, Caroline K. S. Shea, Kelly Y. C. Lai, Suk Ling Ma, Se Fong Hung, Sandra Chan, Thomas N. Y. Kwong, Sunny Wong, Patrick W. L. Leung

**Affiliations:** 1 Department of Psychiatry, The Chinese University of Hong Kong, Hong Kong, China; 2 Department of Psychiatry, Tai Po Hospital, Hong Kong, China; 3 Department of Psychiatry, Alice Ho Miu Ling Nethersole Hospital, Hong Kong, China; 4 Department of Medicine and Therapeutics, The Chinese University of Hong Kong, Hong Kong, China; 5 Department of Psychology, The Chinese University of Hong Kong, Hong Kong, China

**Keywords:** Autism spectrum disorders, Functional dyspepsia, Paediatric research

## Abstract

Emerging evidence of an altered gut microbiome in autism spectrum disorder (ASD) suggests a pathomechanism through the gut–brain axis despite the inconsistent microbiome profile reported across studies. One of the knowledge gaps in the existing ASD microbiota studies is the lack of systematic exploration of the role of comorbid functional gastrointestinal disorder (FGID) in the association of ASD and altered gut microbiome. Consequently, 92 ASD and 112 age-matched typically developing (TD) boys were profiled on general psychopathology, FGID status by Rome IV classification, and gut microbiota using 16S ribosomal RNA amplicon sequencing at the V4 hypervariable region. Compared to TD, a significant decrease in the within-sample abundance of taxa was observed in ASD, regardless of FGID status. The microbiota of ASD FGID+ and ASD FGID− clustered apart from the TD groups. The microbiota of ASD FGID+ also showed qualitative differences from that of ASD FGID− and had the highest-level *Firmicutes: Bacteroidetes* ratio, which was paralleled by elevated levels of anxiety and overall psychopathology. The altered gastrointestinal microbiota composition in ASD appeared to be independent of comorbid FGID. Further studies should address how FGID may mediate neuropsychiatric symptoms in ASD through inflammation along the microbiota–gut–brain axis.

## Introduction

Autism spectrum disorder (ASD) is a neurodevelopmental disorder encompassing impairing neuropsychological and behavioral symptoms, as well as comorbid medical disorders^1^. Gastrointestinal (GI) symptoms are common in ASD^2^, with up to 30.5% of the clinical ASD population suffering from one functional gastrointestinal disorder (FGID)^3^, in which altered gut microbiota composition is implicated as part of the pathophysiology^4^. This is significant, as the microbiota–gut–brain axis postulated bidirectional effects between the GI and the central nervous system to explain the pathophysiology of psychiatric disorders^5^, including ASD^6^. During neurodevelopment, GI microbiota plays a crucial role during brain maturation^7^. It has been shown that within-sample diversity, that is, the alpha diversity of GI microbiota, was associated with the variability of brain connectivities in human infants to mediate emotionality^8^. Germ-free mice displayed behavioral traits that resembled autism^9^. Hence, the gut microbiome could contribute to the pathogenesis of ASD. Furthermore, as ASD children with comorbid FGID have a higher severity of rigid–compulsive behavior^10^, sensory over-responsivity^11^, and anxiety, even at prepubertal ages^12^, the altered gut microbiome in FGID may also mediate neuropsychiatric and behavioral symptoms.

The promising framework of the microbiota–gut–brain axis, coupled with technological advances in next-generation sequencing, has fueled the blooming of gut microbiota research in ASD over the past decade. It has been repeatedly shown that the microbiota composition in ASD children is divergent from that of typically developing (TD) children^13^. However, as published studies were heterogeneous in their study designs regarding participants’ characteristics, such as age, puberty, and gender, specific replicable microbial signatures have not yet been identified. Another major unresolved issue is the complex inter-relationships between the gut microbiome, FGID, and ASD symptoms. It remains inconclusive whether the change in gut microbiota associated with ASD is merely driven by the presence of FGID, as many studies did not characterize FGID^14^ or rigorously identify FGID according to standardized criteria^15–17^, while some studies grouped ASD with or without GI problems together for comparison^18–22^. Among the few studies that compared the microbiome of ASD with and without FGID, a study by Son et al. did not replicate the microbiotic dissimilarity between ASD and TD^23^, which may be due to the use of neurotypical siblings as a comparison group, where the heritability of the autism trait^24^ and the microbiome^25,26^ within the family could not be controlled. To our knowledge, limited studies directly compare the gut microbiome of ASD with and without FGID, despite its potential mediating effect on the severity of clinical symptoms.

Thus, the present study aimed to study the complex relationship between FGID, gut microbiota, and the clinical symptoms of ASD by comparing the gut microbiota in ASD and TD characterized for the presence or absence FGIDs and the severity of general psychopathology, with stringent criteria set to homogenize potential confounding factors on the gut microbiome. The primary objective was to investigate whether altered microbiota composition occurs in ASD in both the presence (FGID+) and absence (FGID-) of FGID. With the association of comorbid FGID with ASD’s clinical symptom severity, the secondary goal was to compare the gut microbiomes of ASD FGID+ and ASD FGID−. We hypothesize that ASD children have an altered microbial composition, in contrast to TD children, regardless of the presence of FGID, and that the comorbidity of FGID further alters the microbial composition in ASD.

## Results

### Demographics and clinical characteristics of the study population

A total of 92 ASD and 112 TD boys were recruited and completed the study. Ages were comparable between the groups, with means of 8.43 (SD 1.54) years and 8.12 (SD 1.99) years, respectively. The maternal age was significantly older in the ASD group, while the paternal age was similar between groups. Parental educational levels were not significantly different, suggesting a similar socioeconomic status between ASD and TD^27^ (Supplementary Table S1). Comparable to a local prevalence study^28^, 54.3% of the ASD participants had comorbid attention deficit hyperactivity disorder (ADHD), and 5.4% had a specific learning disorder (SLD). In addition, 43.5% of the ASD participants were taking at least one psychiatric medication, which includes methylphenidate or atomoxetine (43.5%), aripiprazole or risperidone (10.9%) and cyproheptadine (6.5%). Moreover, 62 participants were classified as FGID+ according to the Rome IV Diagnostic Questionnaires for Paediatric Functional Gastrointestinal Disorders: Parent-report Form for Children (R4PDQ), with 30 (32.6%) in the ASD group and 32 (28.6%) in the TD group. Among all the FGID+ participants, functional abdominal pain disorders (FAPD) was the most common subcategory of FGIDs (14.7%), followed by functional defecation disorders (FDD) at 12.7% and functional nausea and vomiting disorders (FNVD) at 2.9%. None of the participants was classified as having non-retentive faecal incontinence, an entity categorized under FDD other than functional constipation. Hence, participants classified as FDD were all suffering from functional constipation. The distribution of these subcategories of FGIDs is comparable between the ASD group and TD group [χ^2^ = 1.619, p = 0.655], allowing a valid contrast of the gut microbiota between ASD FGID+ and TD FGID+. Body mass index (BMI) were comparable across the four groups of ASD/TD FGID+/− [p = 0.325]. Regarding psychopathology, Autism Spectrum Quotient-10 (AQ-10), Spence Children's Anxiety Scale-Parent Version (SCAS-P) and Strengths and Difficulties Questionnaire-Parent Version (SDQ) were all significantly higher in ASD, with ASD FGID+ having the highest anxiety level and overall psychopathology, followed by ASD FGID− and then the TD groups [p-values all < 0.001]. ASD FGID+ had the lowest fiber intake, while TD FGID− had the highest main nutrient. The demographic, dietary, and clinical characteristics of the four groups are summarized in Table 1. Supplementary Table S2 summarizes the comparisons of the dietary components between the four groups.

**Table 1:** Descriptive of subjects’ demographic, clinical characteristics, dietary patterns, and FGID classification.

	ASD FGID+				ASD FGID−		TD FGID+		TD FGID−		Statistical comparison	
	N (%)			M (SD)	N (%)	M (SD)	N (%)	M (SD)	N (%)	M (SD)	Statistics	p
**Subjects’ characteristics**												
Male	30 (100)			–	62 (100)	–	32 (100)	–	80 (100)	–	–	–
Age	–			8.2 (1.846)	–	8.548 (1.375)	–	8.469 (1.759)	–	7.975 (2.068)	F (3, 81.332) = 1.397	0.25
**Comorbidity**											–	**–**
ADHD	19 (63.3)			–	31 (50)	–	0 (0)	–	0 (0)	–		
SLD	2 (6.7)			–	3 (4.8)	–	0 (0)	–	0 (0)	–		
AQ–10	–			6.733 (1.818)	–	6.516 (2.014)	–	2.906 (1.304)	–	3.05 (1.066)	F(3, 75.043) = 80.399	**< 0.001**
SCAS–P	–			27.67 (14.03)	–	22.76 (10.2)	–	17.03 (8.982)	–	15.88 (9.896)	F(3, 80.483) = 9.625	**< 0.001**
SDQ	–			20.03 (5.067)	–	16.2 (5.075)	–	12.23 (4.193)	–	9.91 (4.109)	F(3, 78.153) = 40.93	**< 0.001**
Taking psychiatric medication (≥ 1)	14 (46.7)			–	26 (41.9)	–	0 (0)	–	0 (0)	–	–	–
Methylphenidate or Atomoxetine	14 (46.7)			–	24 (38.7)	–	0 (0)	–	0 (0)	–		
Aripiprazole or Risperidone	5 (16.7)			–	5 (8.1)	–	0 (0)	–	0 (0)	–		
Cyproheptadine	2 (6.7)			–	4 (6.5)	–	0 (0)	–	0 (0)	–		
BMI	–			15.67 (3.282)	-	16.73 (4.654)	-	17.56 (5.07)	-	16.96 (4.727)	F(3, 75.7) = 1.176	0.325
**Dietary pattern**												
Unhealthy diet	–			1.467 (0.86)	–	1.565 (1.002)	–	1.531 (1.107)	–	1.212 (1.04)	F(3, 83.983) = 1.6	0.196
Fibre intake	–			0.4 (0.563)	–	0.677 (0.566)	–	0.594 (0.56)	–	0.738 (0.568)	F(3, 82.682) = 2.712	**0.05**
Main nutrient intake	–			4.433 (1.251)	–	4.452 (1.387)	–	4.375 (1.1)	–	5.05 (1.066)	F(3, 81.377) = 4.824	**0.004**
**FGID classification**												
FNVD	4 (13.3)			–	–		2 (6.3)	–	–		χ^2^ = 1.619	0.655
FAPD	15 (50)			–	–		15 (46.9)	–	–			
FDD	11 (36.7)			–	–		15 (46.9)	–	–			

Significant values are in bold.

*ASD* Autism Spectrum Disorder, *TD* Typically Developing, *FGID* Functional Gastrointestinal Disorder, *ADHD* Attention Deficit Hyperactivity Disorder, *SLD* Specific Learning Disorder, *AQ-10* Autism Spectrum Quotient 10 items, *SCAS-P* Spence Children's Anxiety Scale—Parent Version, *SDQ* Strengths and Difficulties Questionnaire—Parent Version, *BMI* Body Mass Index, *FNVD* Functional Nausea and Vomiting Disorders, *FAPD* Functional Abdominal Pain Disorders, *FDD* Functional Defecation Disorders.

### Gut microbial diversity between ASD and TD with and without FGID

A total of 12,298,104 high-quality reads and 2,645,778 sequences were obtained from the 204 participants, with an average of 12,969 sequences per sample. Moreover, 3162 features were identified in all samples at a 99% similarity level, with an average of 836 features observed in each sample. Alpha diversity analysis revealed that the within-subject species diversity and abundance were significantly lower in ASD, as measured by both Faith’s Phylogenetic Diversity (Faith’s PD) [p_adjusted_ = 0.001] and the Chao1 Index [p_adjusted_ < 0.001] (Table 2 and Supplementary Fig. S1). For beta diversity, the overall gut microbial composition of ASD and TD was significantly different, as measured by the Bray–Curtis dissimilarity [p_adjusted_ = 0.001], unweighted UniFrac distance metrics [p_adjusted_ = 0.001], and weighted UniFrac distance metrics [p_adjusted_ = 0.001] (Table 3 and Supplementary Fig. S2).

**Table 2:** Summary of Mann–Whitney U tests on alpha diversity indices across ASD and TD groups.

Alpha diversity index	Mean ± SD		p	p (adjusted)	Effect size
	ASD	TD			
Faith’s PD	7.73 (1.65)	8.17 (1.05)	0.020	**0.004**	**0.31**
Chao1 Index	79.8 (19.3)	92.6 (20.2)	< 0.001	**< 0.001**	**0.383**

	Mean ± SD		p	p (adjusted)	Effect size
	ASD FGID−	TD FGID−			
Faith’s PD	7.46 (1.51)	8.12 (1.06)	0.003	**< 0.001**	**0.371**
Chao1 Index	79 (19.4)	92.9 (21.3)	< 0.001	**< 0.001**	**0.396**

	Mean ± SD		p	p (adjusted)	Effect size
	ASD FGID+	TD FGID+			
Faith’s PD	8.27 (1.82)	8.29 (1.03)	0.953	0.496	0.213
Chao1 Index	81.4 (19.4)	91.8 (17.4)	0.030	**0.040**	**0.347**

	Mean ± SD		p	p (adjusted)	Effect size
	ASD FGID+	ASD FGID−			
Faith’s PD	8.27 (1.82)	7.46 (1.51)	0.027	**0.032**	**0.285**
Chao1 Index	81.4 (19.4)	79 (19.4)	0.577	0.487	0.065

	Mean ± SD		p	p (adjusted)	Effect size
	TD FGID+	TD FGID−			
Faith’s PD	8.29 (1.03)	8.12 (1.06)	0.451	0.868	0.143
Chao1 Index	91.8 (17.4)	92.9 (21.3)	0.811	0.407	0.02

Significant values are in bold.

ASD: Autism Spectrum Disorder, TD: Typically Developing; Faith’s PD: Faith’s Phylogenetic Diversity. Statistical comparisons were adjusted for usage of psychiatric medications, unhealthy diet, fibre intake and main nutrient intake; Effect sizes were calculated using rank biserial correlation.

**Table 3:** Summary of PERMANOVA on beta diversity indices across ASD and TD groups.

Comparison			df	Bray–Curtis dissimilarity			Unweighted UniFrac distance			Weighted UniFrac distance		
Group 1	Group 2	Factor		F	p	p (adjusted)	F	p	p (adjusted)	F	p	p (adjusted)
ASD	TD	Group	1	2.899	< 0.001	**< 0.001**	4.510	< 0.001	**< 0.001**	8.064	< 0.001	** < 0.001**
		residuals	202									
ASD FGID−	TD FGID−	Group	1	2.472	< 0.001	**< 0.001**	3.559	< 0.001	**< 0.001**	6.692	< 0.001	**0.001**
		residuals	140									
ASD FGID+	TD FGID+	Group	1	1.427	0.028	**0.035**	2.181	< 0.001	**0.002**	2.268	0.034	**0.042**
		residuals	60									
ASD FGID+	ASD FGID−	FGID	1	1.140	0.21	0.253	1.943	0.016	**0.014**	1.368	0.229	0.194
		residuals	90									
TD FGID+	TD FGID−	FGID	1	1.194	0.187	0.17	1.469	0.043	**0.036**	1.024	0.364	0.372
		residuals	110									

Significant values are in bold.

*ASD* Autism Spectrum Disorder, *TD* Typically Developing, *FGID* Functional Gastrointestinal Disorder. Permutations = 1000 with statistical adjustment for usage of psychiatric medications, unhealthy diet, fibre intake and main nutrient intake.

**Figure 1:**
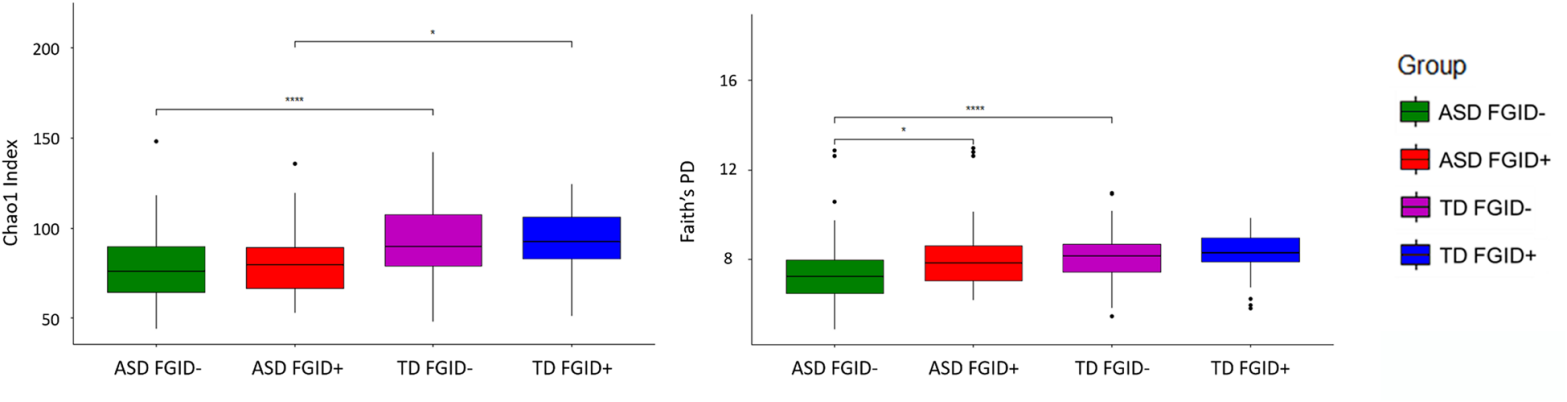
Comparison of alpha diversity (the Chao1 Index and the Faith’s Phylogenetic Diversity) between ASD FGID−, ASD FGID+, TD FGID−, and TD FGID+; *p < 0.05, **p < 0.01, ***p < 0.001, ****p < 0.0001.

Stratifying both ASD and TD into groups of FGID+ or FGID− for pairwise comparison showed that regardless of FGID status, the ASD population had a decrease in the abundance of taxa, measured by the Chao1 index, compared to the corresponding TD groups [FGID−: p_adjusted_ < 0.001; FGID+: p_adjusted_ 0.04], whereas phylogenetic diversity, as measured by Faith’s PD, was lower in ASD in the FGID− [p_adjusted_ < 0.001] but comparable between ASD and TD with FGID. When contrasting ASD with and without FGID, although the Chao1 index was comparable, ASD FGID+ participants had higher Faith’s PD [p_adjusted_ 0.032]. Conversely, the alpha diversity was similar between TD with and without FGID (Table 2, Fig. 1). For beta diversity analysis, the microbial composition was significantly different between ASD FGID- vs. TD FGID- [Bray–Curtis: p_adjusted_ = 0.001; unweighted UniFrac: p_adjusted_ ≤ 0.001; weighted UniFrac: p_adjusted_ = 0.001] and ASD FGID+ vs. TD FGID+ [Bray–Curtis: p_adjusted_ = 0.035; unweighted UniFrac: p_adjusted_ = 0.002; weighted UniFrac: p_adjusted_ = 0.042]. ASD FGID+ vs. ASD FGID− significantly differed in unweighted UniFrac [p_adjusted_ = 0.014], but not in Bray–Curtis dissimilarity and weighted UniFrac distance. A similar pattern was observed in TD with and without FGID [unweighted UniFrac: p_adjusted_ = 0.036]. As unweighted UniFrac measures phylogenetic distances and is sensitive to distinctions in low-abundance features, it follows that for both ASD and TD, the disparity in microbiota composition between those with and without FGID lies in small quantities but phylogenetically distinct microbes (Table 3 and Supplementary Fig. S3).

Given that 43.5% and 54.3% of the ASD participants were taking at least one psychiatric medication and suffering from ADHD respectively, additional groupwise comparisons of 1. Medicated ASD vs Unmedicated ASD, 2. Medicated ASD FGID+ vs Unmedicated ASD FGID+, 3. Medicated ASD FGID− vs Unmedicated ASD FGID−, 4. ASD ADHD+ vs ASD ADHD−, 5. ASD FGID− ADHD+ vs ASD FGID− ADHD−, and 6. ASD FGID+ ADHD+ vs ASD FGID+ ADHD− on all diversity indices were performed. There was no significant difference between medicated and unmedicated ASD in all the comparison groups, lending support to that our main results were not confounded by the usage of psychiatric medications. For ADHD, while most of the indices were comparable between ASD with and without comorbid ADHD, within the ASD FGID− subgroup, ASD with and without ADHD clustered apart in the 3 indices of beta diversity. It follows that the lower alpha diversity in ASD was not confounded by the presence of ADHD, yet comorbid ADHD may have a distinctive microbiota composition within ASD. The additional pairwise analyses were summarized in Supplementary Tables S3 and S4.

Explorative analyses of the diversity indices between ASD and TD on the subtypes of FGID (FNVD, FADP, and FDD) were carried out given their different underlying pathophysiology. However, with the small sample size for each subtype of FGID, no significant difference was noted except for Unweight UniFrac between ASD FDD and TD FDD (Supplementary Tables S5 and S6).

### Differences in taxa between ASD and TD with and without FGID

**Figure 2:**
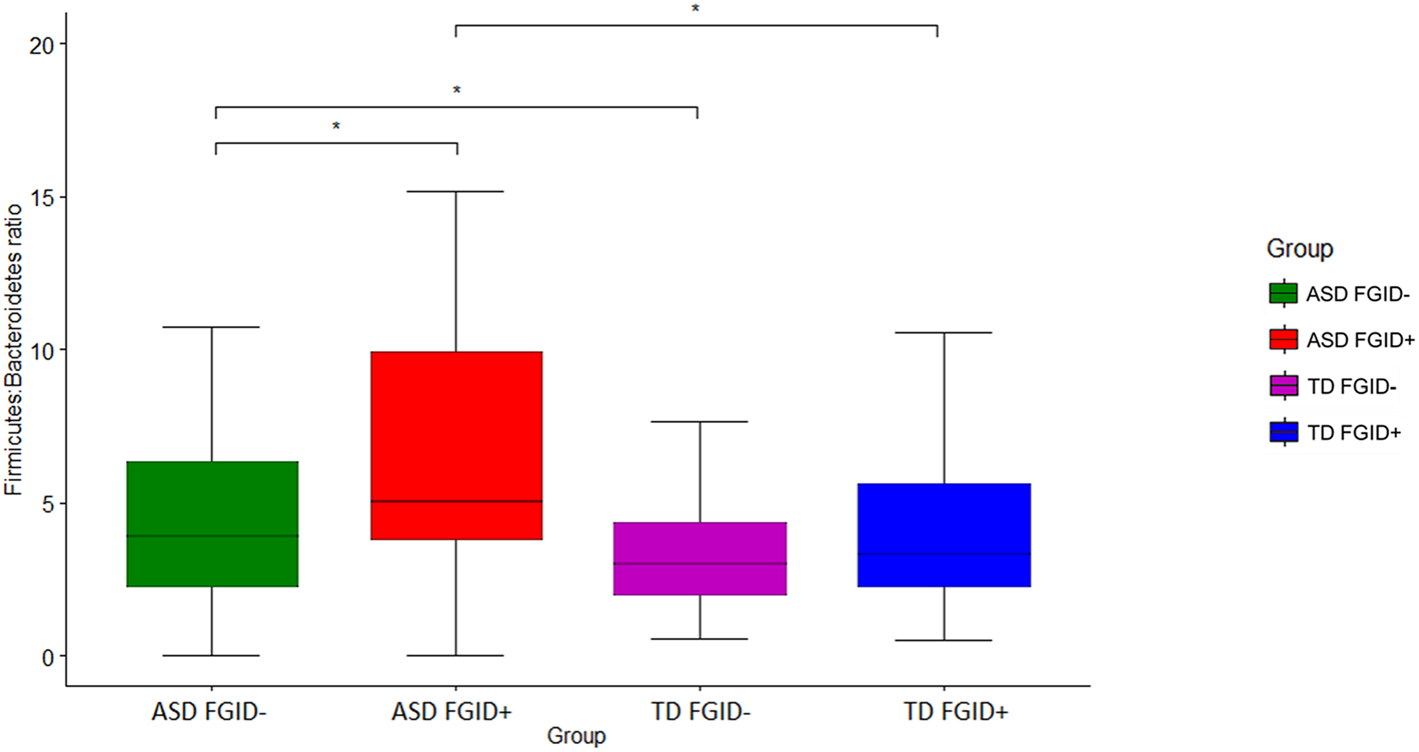
Comparison of the *Firmicutes: Bacteroidetes* ratio between ASD FGID−, ASD FGID+, TD FGID−, and TD FGID+; *p < 0.05, **p < 0.01, ***p < 0.001, ****p < 0.0001.

Further analyses compared the relative abundance of taxa between ASD and TD. *Firmicutes, Bacteroidota, Actinobacteriota, Proteobacteria* and *Desulfobacterota* constituted the most abundant phylum among the participants. Significant variations were observed, and comparisons of the relative abundance at the phylum level between the groups are summarized comprehensively in Supplementary Table S7 and Supplementary Fig. S4. Notably, the *Firmicutes: Bacteroidetes* (F:B) ratio was observed to be higher in ASD than TD [M_ASD_ = 10.9, M_TD_ = 5.78, p = 0.003]. Furthermore, ASD FGID+ had the highest F:B ratio, following a descending trend in ASD FGID− and TD FGID+ to TD FGID− (Table 4, Fig. 2).

**Table 4:** Summary of Mann–Whitney U tests on the *Firmicutes: Bacteroidetes* ratio across ASD and TD groups.

Variable	Mean ± SD		Statistical comparison		
	ASD	TD	Statistics	p	Effect size
*Firmicutes: Bacteroidetes* Ratio	10.9 (30)	5.78 (9.65)	U = 3922	**0.003**	**0.239**
	**Mean ± SD**		**Statistical Comparison**		
	**ASD FGID−**	**TD FGID−**	**Statistics**	**p**	**Effect size**
	7.62 (14.5)	4.9 (8.12)	U = 2002	**0.049**	**0.193**
	**Mean ± SD**		**Statistical Comparison**		
	**ASD FGID+**	**TD FGID+**	**Statistics**	**p**	**Effect size**
	17.7 (48)	7.98 (12.6)	U = 327	**0.031**	**0.319**
	**Mean ± SD**		**Statistical Comparison**		
	**ASD FGID+**	**ASD FGID−**	**Statistics**	**p**	**Effect size**
	17.7 (48)	7.62 (14.5)	U = 691	**0.047**	**0.257**
	**Mean ± SD**		**Statistical Comparison**		
	**TD FGID+**	**TD FGID−**	**Statistics**	**p**	**Effect size**
	7.98 (12.6)	4.9 (8.12)	U = 1125	0.320	0.121

Significant values are in bold.

*ASD* Autism Spectrum Disorder, *TD* Typically Developing, *FGID* Functional Gastrointestinal Disorder. Effect sizes were calculated with rank biserial correlation.

**Figure 3:**
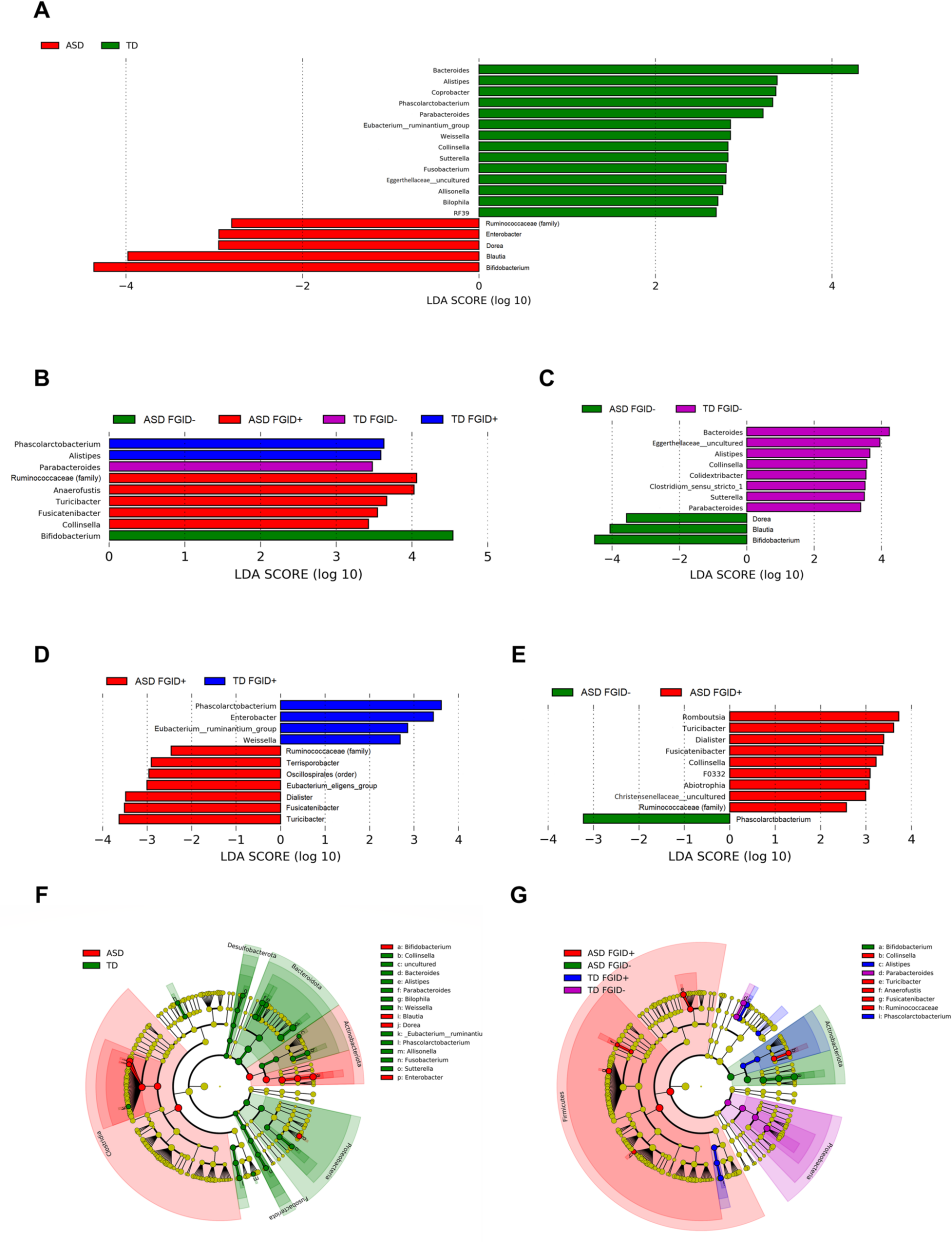
(**A**–**E**) Linear discriminant analysis (LDA) scores for the bacterial taxa that differentiate between (**A**) ASD vs. TD, (**B**) ASD FGID−, ASD FGID+, TD FGID−, and TD FGID+, (**C**) ASD FGID− vs. TD FGID−, D. ASD FGID+ vs/TD FGID+ and (**E**) ASD FGID− vs. ASD FGID+. The colours indicate the taxa enriched in the corresponding group of participants, with only the taxa of LDA score > 2.0 being shown. (**F**,**G**) Cladograms generated by Linear discriminant analysis Effect Size (LEfSe) indicating differences in the bacterial taxa between (**F**) ASD and TD and (**G**) ASD FGID−, ASD FGID+, TD FGID−, and TD FGID+.

The genus-level analysis identified 19 genera that were significantly different between ASD and TD (Supplementary Table S8 and Fig. 3A,F). Discriminative genera were identified in each of the four participant groups (Fig. 3B,G). Compared to TD FGID-, ASD FGID- showed a significant increase in *Bifidobacterium, Dorea,* and *Blautia* and a decrease in *Collinsella*, *Bacteroides, Alistipes, Parabacteroides, Clostridium *sensu stricto* 1, Sutterella*, and *uncultured Eggerthellaceae*. ASD FGID+ was distinctive from ASD FGID- and TD FGID+, with a significant increase in *Ruminococaceae *(*family*), *Dialister, Fusicatenibacter, Turicibacter*, and a decrease in *Phascolarctobacterium* (Table 5, Fig. 3C–E)*.* The comparison between TD FGID+ and TD FGID− is summarized in Supplementary Table S9.

**Table 5:** Comparison of the relative abundance at genus level between ASD and TD groups.

General	Mean ± SD		Statistics		
	ASD FGID−	TD FGID−	p	p _FDR_	Effect size
Bifidobacterium	18.996 (12.85)	12.072 (9.21)	0.001	**0.01**	**0.317**
Bacteroides	13.206 (9.64)	17.085 (10.25)	0.023	**0.026**	**0.223**
Blautia	10.432 (5.98)	7.747 (4.07)	0.008	**0.016**	**0.261**
Clostridium sensu stricto 1	0.933 (2.19)	1.400 (2.29)	0.046	**0.046**	**0.181**
Parabacteroides	0.767 (1.27)	1.291 (1.32)	0.001	**0.005**	**0.313**
Alistipes	0.3472 (0.65)	0.958 (1.355)	0.002	**0.005**	**0.29**
Collinsella	0.215 (0.59)	0.443 (0.61)	0.002	**0.007**	**0.249**
Sutterella	0.075 (0.27)	0.221 (0.49)	0.017	**0.028**	**0.169**
Dorea	0.174 (0.54)	0.028 (0.15)	0.021	**0.026**	**0.109**
Eggerthellaceae uncultured	0 (0)	0.029 (0.12)	0.018	**0.026**	**0.088**

	Mean ± SD		Statistics		
	ASD FGID +	TD FGID +	p	p _FDR_	Effect size
Oscillospirales (order)	20.716 (7.53)	18.518 (9.75)	0.036	0.051	0.133
Ruminococcaceae (family)	18.380 (7.24)	15.662 (8.17)	0.037	**0.046**	**0.214**
Fusicatenibacter	1.440 (0.88)	0.765 (0.68)	< .001	**0.005**	**0.486**
Turicibacter	0.964 (1.35)	0.175 (0.43)	0.001	**0.003**	**0.423**
Dialister	0.917 (1.11)	0.345 (0.60)	0.025	**0.05**	**0.303**
Phascolarctobacterium	0.124 (0.39)	0.897 (1.09)	< 0.001	**< 0.001**	**0.575**
Enterobacter	0.053 (0.18)	0.627 (1.21)	0.033	0.055	0.238
[Eubacterium] eligens group	0.188 (0.54)	0.013 (0.05)	0.024	0.06	0.215
[Eubacterium] ruminantium group	0 (0)	0.124 (0.38)	0.049	0.054	0.125
Weissella	0 (0)	0.013 (0.05)	0.049	**0.049**	**0.125**

	Mean ± SD		Statistics		
	ASD FGID+	ASD FGID−	p	p _FDR_	Effect size
Ruminococcaceae (family)	18.380 (7.24)	15.507 (8.66)	0.002	**0.02**	**0.247**
Fusicatenibacter	1.440 (0.88)	1.016 (1.00)	0.024	**0.048**	**0.289**
Romboutsia	1.6354 (2.92)	0.694 (1.74)	0.03	**0.05**	**0.253**
Dialister	0.917 (1.11)	0.474 (0.86)	0.031	**0.044**	**0.252**
Turicibacter	0.964 (1.35)	0.269 (0.61)	0.002	**0.01**	**0.351**
Collinsella	0.569 (0.77)	0.215 (0.59)	0.003	**0.008**	**0.299**
Phascolarctobacterium	0.124 (0.39)	0.450 (0.90)	0.002	**0.007**	**0.339**
Christensenellaceae uncultured	0.006 (0.02)	0 (0)	0.043	0.054	0.067
F0332	0.004 (0.01)	0 (0)	0.043	**0.048**	**0.067**
Abiotrophia	0.004 (0.02)	0 (0)	0.043	**0.043**	**0.067**

Significant values are in bold.

*ASD* Autism Spectrum Disorder, *TD* Typically Developing, *FGID* Functional Gastrointestinal Disorder. P-values corrected for false discovery rate using the Benjamini–Hochberg method. Effect sizes were calculated with rank biserial correlation. Genera are presented in descending order of relative abundance.

As the ASD groups were characterized by a lower alpha diversity and a higher F:B ratio, correlations between these indices with clinical parameters of AQ-10, SCAS-P and SDQ were also explored. It was observed that the lower alpha diversity and higher F:B ratio were significantly associated with higher AQ-10 and SDQ scores (Supplementary Table S10).

## Discussion

In this case-controlled sample of prepubertal ASD boys and age- and gender-matched TD, the altered microbiota composition was observed in ASD over TD, considering the confounding effect of comorbid FGID. The gut microbiome of ASD comorbid with FGID differed from ASD without FGID by phylogenetically distinct taxa that were of low relative abundance. Furthermore, autistic symptoms (AQ-10), anxiety (SCAS-P) and overall psychopathology (SDQ) levels were highest among the ASD FGID+ and explorative analyses also showed that lower alpha diversity and microbiota composition at the phylum level (F:B ratio) correlated with higher SDQ and AQ-10. Our results thus corroborated the reported potential correlations between microbiota, GI and neuropsychiatric symptoms in ASD^10–12^. Notwithstanding the rigorous attempts made to control for possible confounders and optimize the characterization of psychopathology and FGIDs in this study, our findings should be interpreted from the wider perspective of mixed results in published studies. It is also worth noting that, a higher or lower alpha diversity do not have a direct implication on the host’s health status and should be interpreted as a consequence of the ecological mechanism that drives the observations^29^. Therefore, the compositional changes of microbiota in ASD in the present study, regardless of the directionality, should prompt further investigation on the underlying biological cause. Further, the degree and pattern of disease-associated microbiota changes vary across different entities, with enrichment of a small quantity of pathogens, depletion of health-related taxa and gross compositional changes being reported^30^, and little is known in this area for ASD.

### Alteration of gut microbiota composition in ASD: merely a confounding effect of FGID or an independent signature?

There were inconsistent results across alpha diversity analyses in ASD, where a decrease, increase, and most commonly, similar richness and diversity were reported^31^. Our results showed that, when excluding FGID, a homogenous decrease in alpha diversity across the two indices measured was observed in ASD. Also, beta diversity showed the microbiota in ASD are distinct from TD in subjects without FGID across the three indices measured. Collectively, these findings suggest that the altered abundance, diversity, and structure of microbiota in ASD is not merely a result of the higher prevalence of comorbid FGID.

Within our sample, several genera were found to be significantly different and discriminative between ASD and TD without FGID. However, some of the results conflicted with a recent systematic review^13^. For example, *Bifidobacterium*^32–35^ and *Blautia*^36,37^ were both reported to have a lower relative abundance in ASD, while an increase in *Collinsella*^38^ was observed. Mixed results (both increase and decrease) were also reported for *Dorea, Bacteroides, Alistipes, Oarabacteroides,* and *Sutterella*^13^. Here, several possible reasons are discussed for the inconsistencies in methodological heterogeneity among the studies included in the systematic review. First, some studies quoted either did not separate ASD participants with and without GI symptoms or FGID prior to analysis^33,38^, or included only ASD participants with comorbid GI symptoms^32,36,37^. Second, the gender distribution between ASD and the controls was unequal for some studies^33–36^, with females being over-represented in the control group. This may confound the results, given that *Bifidobacterium* were reported to be higher in females^39^. Third, compared to the present study, the sample sizes of these previous studies were all relatively smaller, in the range of six to 40 ASD cases. Despite so, the result of increase in abundance of *Bifidobacterium* in ASD FGID− relative to TD FGID− warrants further investigation, as the genus is frequently cited as beneficial^40^.

Altered gastrointestinal microbiota composition is implied in the pathophysiology of ASD. It has been recently shown that host genetics and gut microbiota interact to regulate immune and metabolic pathways relevant to ASD^41^, and various pathways have been postulated on how the microbiome can exert its influence on the host’s brain. These include the regulation of gut permeability through tight junction protein, the production of short-chain fatty acids (SCFAs), and the metabolism of tryptophan and inflammatory cytokines^7^. Within the genera that discriminated between FGID- ASD and TD from the present study, *Alistipes, Bifidobacterium, Blautia,* and *Collinsella* are all fermenters that produce SCFAs, while *Dorea* and *Sutterella* could regulate the mucosal metabolism to affect gut permeability^42,43^. With a lower relative abundance of *Bacteroides* in the ASD FGID− group observed, it has been shown that oral treatment with *Bacteroides fragilis* corrected ASD-related behavior in mice by altering gut permeability and metabolic pathways^44^.

### Is ASD FGID+ a distinctive subgroup in the gut microbiome profile?

Within the smaller sample of FGID+ participants, the lower Chao1 index and beta diversity analysis indicated that the microbiota of ASD FGID+ had a lower taxa abundance and were clearly clustered apart from that of TD FGID+. Although there are distinct pathophysiologies of each FGID subtype^45^, given the comparable distribution of FGID subtypes in the ASD and TD groups in this study, our observations are unlikely to be confounded by the biological factors unique to individual subtypes of FGID. Hence, the altered gut microbiota composition in ASD FGID+ did not appear to be solely attributable to FGID, but possibly reflect an additive effect of both ASD-associated and FGID-associated changes in the microbiome.

Furthermore, with higher Faith’s PD in the ASD FGID+ group relative to ASD FGID−, and the two groups clustered only in the measurement of unweighted UniFrac distance but not weighted UniFrac and Bray Curtis dissimilarity, it appears the microbiota of ASD FGID+ was phylogenetically more diverse, and these phylogenetically distinct taxa that presented in low relative abundance were setting ASD FGID+ apart from ASD FGID−. Comparatively, the distinction between ASD FGID+ and ASD FGID− was not as pronounced as when ASD was compared with TD. Hence, the microbiota of ASD with and without FGID could lie along a continuum within the altered gut microbiota composition of ASD.

As an increase in the F:B ratio was suggested to be linked with a proinflammatory state, there was a gradient of increase of the F:B ratio from TD FGID−, ASD FGID−, and TD FGID+ to the highest in the ASD FGID+ participants. Intriguingly, this gradient ratio also paralleled the clinical observation that the ASD FGID+ group had the highest levels of anxiety and overall psychopathology. Thus, the correlation between FGID and neuropsychiatric symptoms in ASD could be mediated by a proinflammatory state along the microbiota–gut–brain axis that warrants further exploration.

Among the genera that distinguished ASD FGID+ from both ASD FGID− and TD FGID+, *Fusicatenibacter, Dialister*, and *Phascolarctobacterium* are producers of SCFAs. Our results also showed that ASD FGID+ was enriched with *Turicibacter,* the abundance of which was found to have a unique reciprocal positive effect on the gut luminal level of serotonin (5-HT)^46^. *Turicibacter*, together with other spore-forming bacteria dominated by the *clostridia* class, promote the gut production of 5-HT by inducing the rate-limiting enzyme tryptophan hydroxylase-1, while 5-HT conversely promotes the competitive colonization of *Turicibacter* in the intestine. Gut-derived 5-HT, which constitutes > 90% of the human body’s 5-HT^47^, is an important modulator of gastrointestinal motility^48^, and an altered level of 5-HT in the gastrointestinal system was implicated in the pathophysiology of FGIDs^49^. Thus, the increase in the relative abundance of *Turicibacter* observed in our ASD FGID+ participants may illuminate the pathophysiology of GI symptoms in ASD individuals. Furthermore, the 5-HT produced in the gut is stored in circulating platelets and may result in hyperserotonaemia, a phenomenon observed in 30% of ASD cases^50^. While the phenomenon remained enigmatic in its clinical correlates and causes, our results revealed the altered gut microbiota in ASD may provide clues to its occurrence.

## Future directions and limitations

Although the present study is insufficient to infer or elucidate the underlying pathophysiological mechanism of microbiota changes in ASD, the clarification of their relationship with FGID serves as a starting point to further understand the causes and effects in young children. Direct measurement of the functional output of the gut microbiome through a multi-omics approach, including metatranscriptomics, metabolomics, and metaproteomics, could further reveal the downstream effect of microbiota to infer the possible pathomechanism^51^. In our present study, a functional analysis such as PICRUSt^52^ was not conducted for two main reasons. First, the main objective was to verify the change in the microbiota composition in ASD independent of comorbid FGID rather than to propose any possible pathomechanism. Second, there is an inherent limitation of 16 s amplicon sequencing in terms of the taxonomic assignment beyond the genus level and thus the accuracy of functional analysis^53^. Beyond that, the cross-talk between the microbiome and the host’s genetic expression is also worth investigating as a potential pathogenesis pathway^54^.

ASD is heterogeneous in its severity, which is multi-dimensionally captured by the core ASD symptoms, comorbid psychopathology and functional level^55^, and whether gut microbiota has a role in the severity of core symptoms and comorbid psychopathology in ASD is scarcely addressed. Our explorative analyses showed that microbiota composition correlates with these clinical aspects of ASD, and within the ASD FGID− subgroup, microbiota was distinctive between those with and without ADHD. A systematic investigation by profiling the ASD symptom domains and comorbid psychopathology comprehensively, and comparisons with non-ASD individuals with similar psychopathology, would shed light on the potential mediating and moderating role of gut microbiota in the diverse clinical phenotypes of ASD. The medications’ effect on gut microbiota composition in ASD is also to be studied by considering individual medication and the dosage effect.

Several methodological limitations should be considered when interpreting the findings of this study. First, while a stringent sampling frame was implemented to minimize the confounding effects of puberty and gender, this would also limit the generalizability of the results. Second, some stool samples stored overnight at the participants’ domestic refrigerator at 4 °C were not ideal and could introduce spurious results. Third, the dietary assessment captured merely an overall food composition, which lacked information on specific food types and allergy, dietary preference, and history of dietary intervention. Comprehensive profiling of these would help uncover potential mediating and moderating effects on the gut microbiota by diet, as suggested in a recent study^56^. Fourth, the sample size of FGID+ was relatively small, which is a heterogeneous group of disorders with divergent pathophysiology. The different stool transit and fermentation time in constipation and diarrhoea may result in different bacterial composition. Hence, categorizing different FGIDs as a single group in the analyses has inevitably precluded the study of biodiversity across the different types of FGIDs. A sufficiently powered study with the full range of FGIDs would be needed in the future to investigate their specific associations with the gut microbiome in ASD. Also, there was a lack of dimensional measurement of GI symptoms to consider the spectrum of varying FGID severity and overlapping symptoms^57^. Finally, the evaluation of the anxiety level, psychopathological severity, and FGID status was respondent-based, which may introduce response biases.

## Conclusion

The presence of altered gut microbiota composition was associated with ASD after accounting for the presence of FGID. A small quantity of phylogenetically distinct taxa characterized the differences in the gut microbiome between ASD with and without FGID. Further understanding of the pathomechanism in the microbiota–gut–brain axis in ASD would benefit from functional analyses of the gut microbiome and the utilization of a multi-omics approach in combination with network analysis.

## Methods

### Recruitment and clinical assessment of research participants

In this case–control study, Chinese ASD and TD boys aged between 4 and 11 years old were recruited. Other than the presence of FGID in the FGID+ groups, all participants did not have other major illnesses such as organic GI or neurological disorders. The age range of the participants was confined to 4–11 years old to minimize the effect of puberty^58^, while ensuring the structural stability of the microbial community, which is generally reached at 3 years of age^59^. Only boys were studied, as the effect of microbiota on the brain could be gender-dependent^8^. The study was approved by the Joint Chinese University of Hong Kong—New Territories East Cluster Clinical Research Ethics Committee (Ref no.: CRE-2016.700). ASD boys were recruited from a child and adolescent psychiatric outpatient clinic at a public sector hospital with a diagnosis made according to the Diagnostic and Statistical Manual of Mental Disorders, Fifth Edition (DSM-5) by child psychiatrists with post-specialist qualifications. The clinic is the sole provider of public child and adolescent psychiatric services in the catchment area covering one-sixth of the population in Hong Kong. Community-based TD children with reported good general physical and mental health were recruited from public schools. Participants and their parents were informed of the details of the study, and written informed consent was obtained from the parents according to the Declaration of Helsinki. Clinical records were reviewed for ASD boys, and demographic data were obtained from the parents of all the participants. Body height and weight were measured to calculate their BMI. All parents were asked to complete the following questionnaires: (1) The R4PDQ, an instrument designed to assess FGID in children in both clinical and research settings according to the Rome IV diagnostic criteria^45^. From the three broad categories of FGID, namely FNVD, FAPD and FDD, 10 specific disorders could be identified. The R4PDQ was validated against a gastroenterologist’s diagnosis and demonstrated diagnostic accuracy as a respondent-based classification instrument^60^. The R4PDQ was translated into Chinese and back-translated into English by independent bilingual members of our research group. A panel of bilingual gastroenterologists compared the original English version with the back-translation, and discrepancies were identified and resolved. The final translated version was approved by the committee of the Rome Foundation; (2) The AQ–10, a 10-item, parent-report brief version of the Autism Spectrum Quotient to exclude potential ASD cases in community-based children^61^; (3) The Chinese version of SDQ, a 25-item questionnaire that measures overall psychopathology in children. A total score of < 17 carries a negative predictive value of 93% for the presence of mental disorders based on local prevalence^62^; (4) The Chinese version of the SCAS-P^63^, a 38-item questionnaire that captures the severity of anxiety symptoms, a common comorbid condition in FGID and in ASD; and (5) A dietary questionnaire developed by the Department of Health of the Hong Kong SAR government, which was used in a community child health survey in 2005–2006 in Hong Kong^64^. Parents completed the questionnaire on children’s eating behaviors and dietary habits to obtain information on their main nutrient intake (the amount of milk, fruit, vegetables, cereal, meat, fish, beans and egg consumed), fiber intake (the amount of fruit and vegetables consumed), and unhealthy intake (the amount soft drink and fried food consumed, habit of eating at fast food and fatty meat) over the past seven days. Details of the dietary questionnaire and the algorithm for calculating the three composite scores is listed in the Supplementary Information. The exclusion criteria were: (1) A score above the cut-off (≥ 6) on the AQ–10 for the TD group^61^; (2) Intellectual disability and known history of organic GI disorder based on the medical record; (3) The use of antibiotics within a month before stool sample collection; and (4) The regular daily use of probiotics within a month prior to stool sample collection.

### Stool sample collection, microbial DNA extraction, and 16S rRNA gene sequencing

Approximately 200–300 mg of stool samples were collected from each participant at home by their parents. Verbal and written instructions were given to the parents to avoid contamination of the stool samples. The stool samples collected in sterile tubes without preservatives were transferred immediately to the laboratory. If the sample was obtained beyond office hours, it would be stored overnight in a domestic refrigerator at 4 °C before immediate transferral on the next day. All samples reached the laboratory within 24 h and were stored at − 80 °C before DNA extraction and 16S ribosomal RNA (rRNA) gene sequencing. Microbial genomic DNA was extracted from stool samples using the Zymo Research DNA Miniprep Kit (Zymo Research Corporation, Irvine, CA), following the manufacturer’s protocol. The DNA concentration and A260/280 ratio were assessed and evaluated using nanodrops for a quality check. Library construction and sequencing were performed by Macrogen Inc. (Seoul, South Korea). DNA was quantified with PicoGreen and assessed using gel electrophoresis before library construction. Then, a 16S rRNA sequence library was prepared using the Herculase II Fusion DNA Polymerase Nextera XT Index Kit V2 on the MiSeq Illumina platform. The barcoded primer targeting the 16S rRNA V4 hypervariable region was used for sequencing. Paired-end reads of 301 bp were obtained with a sequencing depth of 50,000 reads and qualified by the Phred Quality Score.

### Bioinformatics and data processing

Data processing was performed using the QIIME 2 bioinformatics software package^65^. Raw paired FASTQ sequences files were demultiplexed using the q2-demux plugin and controlled for sequence quality with the processing pipeline DADA2 (q2-dada2). The processing of reads with the dada2-paired method was preceded by trimming the first five bases and truncating at 300 bases in all forward and reverse reads. Reads were filtered, dereplicated and merged to generate the following: (1) A feature table containing counts of 16S rRNA gene sequence variants for each sample; (2) A representative sequence table containing the sequences for each unique feature. A taxonomy was then assigned to each feature sequence against the Silva-138 database using the classify-consensus-vsearch implemented in the q2-feature-classifier plugin. Feature tables were further filtered by removing sequences identified as archaea, mitochondria, or chloroplasts. Following the taxonomy assignment, the feature sequences were aligned with the q2-alignment plugin MAFFT. An unrooted tree using the FastTree method and a rooted tree using the midpoint rooting method were generated with the q2-phylogeny plugin. The QIIME 2 output files, including a feature table, taxonomy, and unrooted tree, were imported to R software (Version 4.0.3) to perform further data processing and analysis. Feature tables were rarefied to 6808 sequences per sample.

### Statistical analysis

Demographics of the participants were compared using the Chi-square test for categorical data, the Student t-test, or the Wilcoxon rank-sum test for continuous data, depending on the normality. Both ASD and TD participants were stratified into FGID+ and FGID− subgroups according to the Rome IV diagnostic criteria. An overall comparison between ASD and TD was conducted before subgroup analyses pertaining to our specific study objectives. First, we compared ASD FGID− vs. TD FGID− and ASD FGID+ vs. TD FGID+ to test if ASD has altered microbiomes both in the presence and absence of FGID. Second, we compared ASD FGID+ vs. ASD FGID− for disparities in the microbiome between ASD with and without FGID. TD FGID+ vs. TD FGID− was also conducted as a reference. Several indices were computed using the phyloseq R package to compare the compositionality of the gut microbiome between groups, with each index having its own approach to measure the ecological structure. First, to contrast the compositional complexity between ASD and TD (i.e. within-sample diversity or alpha diversity), the Chao1 index^66^ and Faith’s PD^67^ were estimated and compared between groups with adjusted multivariate linear regression analyses. The Chao1 index is an abundance-based estimator of species richness, while Faith’s PD considers phylogenetic distance when estimating the diversity. Subsequently, to test for the taxonomical differences between ASD and TD (i.e. between-sample diversity or beta diversity), Bray–Curtis dissimilarity^68^, unweighted, and weighted UniFrac^69^ were used. Bray–Curtis dissimilarity quantifies dissimilarity based on abundances of shared taxa, while UniFrac takes the phylogenetic tree into consideration for compositional dissimilarity, with unweighted UniFrac calculated on the mere presence or absence of the taxa and weighted UniFrac calculated on the disparity in the proportional abundance of the taxa. Hence, unweighted UniFrac is more sensitive to differences in low-abundance features. The permutational Multivariate Analysis of Variance (PERMANOVA) was performed on these three indices using the adonis function implemented in the vegan R package with 1000 permutations, which were then visualized using principal coordinates analysis (PCoA). All testing on alpha diversity and beta diversity estimates were performed with adjustments to the usage of psychiatric medications and dietary information as covariates.

After the compositional structures, variations in the relative abundance between the ASD and TD groups at the phylum and genus levels were compared using the Wilcoxon rank-sum test, assuming the data of relative abundance were not normally distributed. The F:B ratio, which is composed of the two most abundant phyla of the human gut microbiome, was measured and compared. An increased F:B ratio was reported in ASD^38^, and inflammatory bowel disease^70^ and was proposed to be related to inflammatory status^71^. P-values were corrected for the false discovery rate (FDR) upon multiple comparisons using the Benjamini–Hochberg method. Finally, the Linear discriminant analysis Effect Size (LEfSe) analysis was performed using the online Galaxy Hutlab LEfSe platform^72^. Kruskal–Wallis tests within the LEfSe were implemented to analyze all features to identify the differentially abundant features that distinguish the groups, with the threshold of statistical significance at 0.05 and the logarithmic linear discriminant analysis (LDA) score for discriminative features at 2.0.

## Supplementary Information

Supplementary Information.

## Data Availability

The datasets generated during and/or analysed during the current study are available from the corresponding author on reasonable request.
