# Supplementary material for: Disentangling the relationship of gut microbiota, functional gastrointestinal disorders and autism: a case–control study on prepubertal Chinese boys
Source: Sci Rep. 2022 Jun 23;12:10659. doi: 10.1038/s41598-022-14785-8 (PMC9225987; doi:10.1038/s41598-022-14785-8)
Supplement: Supplementary file 1 — Supplementary Information. [file 41598_2022_14785_MOESM1_ESM.docx]

| **Table S1**  Descriptives of participants’ parental demographic | | | | | | | | | | | | | | |
| --- | --- | --- | --- | --- | --- | --- | --- | --- | --- | --- | --- | --- | --- | --- |
|  |  | **ASD (n = 92)** | | | |  | **TD (n = 112)** | | | |  | **Statistical Comparison** | | |
|  |  | **N (Missing)** | **%** | **M** | **SD** |  | **N (Missing)** | **%** | **M** | **SD** |  | **Statistics** | **p** |  |
| ***Parents characteristics*** | | | | | | | | | | | | | | |
| Father's age | | 87 (5) | - | 45.8 | 6.43 |  | 106 (6) | - | 45 | 6.47 |  | U = 4357 | 0.51 |  |
| Father's education | |  |  |  |  |  |  |  |  |  |  | χ² = 1.61 | 0.446 |  |
|  | Primary | 3 | 3.40 | - | - |  | 8 | 7.50 | - | - |  |  |  |  |
|  | Secondary | 46 | 52.90 | - | - |  | 55 | 51.90 | - | - |  |  |  |  |
|  | Tertiary | 39 | 44.80 | - | - |  | 43 | 40.60 | - | - |  |  |  |  |
|  | Unknown | 4 | 4.60 | - | - |  | 6 | 5.70 | - | - |  |  |  |  |
| Mother's age | | 89 (3) | - | 41.2 | 4.26 |  | 108 (4) | - | 39.3 | 4.61 |  | U = 3722 | **0.006** |  |
| Mother's education | |  |  |  |  |  |  |  |  |  |  | χ² = 0.355 | 0.837 |  |
|  | Primary | 5 | 5.60 | - | - |  | 5 | 4.60 | - | - |  |  |  |  |
|  | Secondary | 50 | 56.20 | - | - |  | 59 | 54.60 | - | - |  |  |  |  |
|  | Tertiary | 33 | 37.10 | - | - |  | 45 | 41.70 | - | - |  |  |  |  |
|  | Unknown | 4 | 4.50 | - | - |  | 3 | 2.80 | - | - |  |  |  |  |
| Note. ASD: Autism Spectrum Disorder; TD: Typically Developing | | | | | | | | | | | | | | |

| **Table S2**  One-Way ANOVA (Welch's) comparison on the components of the dietary questionnaire between ASD and TD groups | | | | | | | | | |
| --- | --- | --- | --- | --- | --- | --- | --- | --- | --- |
|  |  | **Mean (SD)** | | | |  |  |  |  |
|  |  | ASD FGID+ | ASD FGID- | TD FGID+ | TD FGID- | **F** | **df1** | **df2** | **p** |
| Main nutrients and Fibre intake | |  |  |  |  |  |  |  |  |
|  | Milk | 0.133 (0.346) | 0.177 (0.385) | 0.031 (0.177) | 0.163 (0.371) | 3.243 | 3 | 90.956 | 0.02563 |
|  | Fruit | 0.367 (0.49) | 0.613 (0.491) | 0.531 (0.507) | 0.675 (0.471) | 3.081 | 3 | 81.501 | 0.03196 |
|  | Vegetables | 0.033 (0.183) | 0.065 (0.248) | 0.063 (0.246) | 0.075 (0.265) | 0.302 | 3 | 87.614 | 0.82386 |
|  | Cereal | 0.467 (0.507) | 0.419 (0.497) | 0.375 (0.492) | 0.512 (0.503) | 0.73 | 3 | 82.523 | 0.53686 |
|  | Meat | 1 (0) | 0.984 (0.127) | 0.969 (0.177) | 1 (0) | - | 3 | - | - |
|  | Fish | 0.933 (0.254) | 0.823 (0.385) | 0.844 (0.369) | 0.938 (0.244) | 1.801 | 3 | 79.361 | 0.15382 |
|  | Beans | 0.533 (0.507) | 0.484 (0.504) | 0.625 (0.492) | 0.688 (0.466) | 2.2 | 3 | 81.37 | 0.09429 |
|  | Egg | 0.967 (0.183) | 0.887 (0.319) | 0.938 (0.246) | 0.988 (0.112) | 2.131 | 3 | 70.712 | 0.10398 |
|  |  |  |  |  |  |  |  |  |  |
| Unhealthy diet | |  |  |  |  |  |  |  |  |
|  | Soft drink | 0.1 (0.305) | 0.161 (0.371) | 0.219 (0.42) | 0.113 (0.318) | 0.779 | 3 | 81.124 | 0.5088 |
|  | Fast food | 0.533 (0.507) | 0.613 (0.491) | 0.656 (0.483) | 0.375 (0.487) | 3.887 | 3 | 82.147 | 0.01189 |
|  | Snack | 0.067 (0.254) | 0.161 (0.371) | 0.094 (0.296) | 0.1 (0.302) | 0.716 | 3 | 85.902 | 0.54509 |
|  | Fried food | 0.367 (0.49) | 0.355 (0.482) | 0.188 (0.397) | 0.275 (0.449) | 1.339 | 3 | 83.285 | 0.26731 |
|  | Fatty meat | 0.4 (0.498) | 0.274 (0.45) | 0.375 (0.492) | 0.375 (0.487) | 0.762 | 3 | 81.579 | 0.51858 |
| Note. ASD: Autism Spectrum Disorder; TD: Typically Developing; FGID: Functional Gastrointestinal Disorder | | | | | | | | | |

| **Table S3** Summary of Mann Whitney U test on alpha diversity indices within ASD group | | | | | |
| --- | --- | --- | --- | --- | --- |
| Comparisons between medicated and unmedicated ASD subjects | | | | | |
| **Alpha diversity index** | **Mean ± SD** | | **p** | **p (adjusted)** | **Effect size** |
|  | **Medicated** | **Unmedicated** |  |  |  |
| Faith’s PD | 7.919 (1.808) | 7.577 (1.516) | 0.327 | 0.312 | 0.063 |
| Chao1 Index | 79.95 (17.776) | 79.692 (20.6) | 0.959 | 0.838 | 0.007 |
|  |  |  |  |  |  |
|  | **Mean ± SD** | | **p** | **p (adjusted)** | **Effect size** |
|  | **Medicated FGID+** | **Unmedicated FGID +** |  |  |  |
| Faith’s PD | 8.519 (2.161) | 8.053 (1.492) | 0.493 | 0.39 | 0.009 |
| Chao1 Index | 83 (18.7) | 80.062 (20.499) | 0.687 | 0.786 | 0.138 |
|  |  |  |  |  |  |
|  | **Mean ± SD** | | **p** | **p (adjusted)** | **Effect size** |
|  | **Medicated FGID-** | **Unmedicated FGID-** |  |  |  |
| Faith’s PD | 7.596 (1.537) | 7.365 (1.498) | 0.645 | 0.661 | 0.071 |
| Chao1 Index | 78.308 (17.410) | 79.528 (20.933) | 0.748 | 0.856 | 0.049 |
|  |  |  |  |  |  |
| Comparisons between ADHD+ and ADHD- ASD subjects | | |  |  |  |
|  | **Mean ± SD** | |  |  |  |
|  | **ADHD+** | **ADHD-** | **p** | **p (adjusted)** | **Effect Size** |
| Faith’s PD | 7.975 (1.809) | 7.429 (1.399) | 0.114 | 0.095 | 0.031 |
| Chao1 Index | 80.14 (17.438) | 79.405 (21.559) | 0.857 | 0.702 | 0.002 |
|  |  |  |  |  |  |
|  | **ADHD+ FGID-** | **ADHD- FGID-** | **p** | **p (adjusted)** | **Effect Size** |
| Faith’s PD | 7.73 (1.731) | 7.194 (1.212) | 0.164 | 0.244 | 0.022 |
| Chao1 Index | 79.516 (17.914) | 78.516 (21.051) | 0.841 | 0.872 | 0.000 |
|  |  |  |  |  |  |
|  | **ADHD+ FGID+** | **ADHD- FGID+** | **p** | **p (adjusted)** | **Effect Size** |
| Faith’s PD | 8.374 (1.909) | 8.09 (1.72) | 0.687 | 0.274 | 0.035 |
| Chao1 Index | 81.158 (17.063) | 81.909 (23.809) | 0.921 | 0.762 | 0.003 |
| Note. ADHD: Attention Deficit Hyperactivity Disorder ASD: Autism Spectrum Disorder; FGID: Functional Gastrointestinal Disorder; Faith’s PD: Faith’s Phylogenetic Diversity. Statistical comparison was adjusted for unhealthy diet, fibre intake and main nutrient intake. Effect size was calculated using rank biserial correlation. | | | | | |

| **Table S4** Summary of PERMANOVA on beta diversity indices within ASD group | | | | | | | | | | | | |
| --- | --- | --- | --- | --- | --- | --- | --- | --- | --- | --- | --- | --- |
| Comparisons between medicated and unmedicated subjects | | | | | | | | | | | | |
| **Comparison** | | **Bray-Curtis** | | |  | **Unweighted Unifrac** | | |  | **Weighted Unifrac** | | |
| **Group 1** | **Group 2** | **F** | **p** | **p_(adjusted)_** |  | **F** | **p** | **p_(adjusted)_** |  | **F** | **p** | **p_(adjusted)_** |
|  |  |  |  |  |  |  |  |  |  |  |  |  |
| Medicated | Unmedicated | 1.058 | 0.372 | 0.419 |  | 1.376 | 0.062 | 0.186 |  | 1.47 | 0.16 | 0.24 |
| Medicated ASD FGID+ | Unmedicated ASD FGID+ | 0.952 | 0.578 | 0.65 |  | 1.011 | 0.426 | 0.639 |  | 0.46 | 0.884 | 0.782 |
| Medicated ASD FGID- | Unmedicated ASD FGID- | 0.947 | 0.536 | 0.689 |  | 1.259 | 0.11 | 0.33 |  | 1.296 | 0.241 | 0.362 |
|  |  |  |  |  |  |  |  |  |  |  |  |  |
| Comparisons between ADHD+ and ADHD- subjects | | | | | | | | | | | | |
| **Comparison** |  | **Bray-Curtis** | | |  | **Unweighted Unifrac** | | |  | **Weighted Unifrac** | | |
| **Group 1** | **Group 2** | **F** | **p** | **p_(adjusted)_** |  | **F** | **p** | **p_(adjusted)_** |  | **F** | **p** | **p_(adjusted)_** |
| ADHD+ | ADHD- | 1.068 | 0.312 | 0.309 |  | 1.368 | 0.055 | 0.051 |  | 1.750 | 0.124 | 0.121 |
| ADHD+ FGID- | ADHD- FGID- | 1.791 | 0.009 | **0.007** |  | 1.853 | 0.007 | **0.004** |  | 4.649 | 0.003 | **0.001** |
| ADHD+ FGID+ | ADHD- FGID+ | 1.201 | 0.135 | 0.147 |  | 1.160 | 0.211 | 0.182 |  | 0.777 | 0.498 | 0.527 |
| Note. ADHD: Attention Deficit Hyperactivity Disorder; ASD: Autism Spectrum Disorder; FGID: Functional Gastrointestinal Disorder. Permutations = 1000 with statistical adjustment for usage of psychiatric medications, unhealthy diet, fibre intake and main nutrient intake. | | | | | | | | | | | | |

| **Table S5**  Summary of Mann Whitney U test on alpha diversity indices between ASD and TD on FGID subgroups | | | | | |
| --- | --- | --- | --- | --- | --- |
|  | **Mean ± SD** | |  |  |  |
|  | **ASD FNVD** | **TD FNVD** | **p** | **p (adjusted)** | **Effect Size** |
| Faith-PD | 7.481 (0.331) | 8.557 (0.903) | 0.08098 | 0.85488 | 0.03 |
| Chao1 | 73.5 (8.583) | 101.5 (23.335) | 0.07963 | 0.1049 | 0.536 |
|  |  |  |  |  |  |
|  | **ASD FAPD** | **TD FAPD** | **p** | **p (adjusted)** | **Effect Size** |
| Faith-PD | 8.786 (2.197) | 8.115 (1.267) | 0.31361 | 0.52842 | 0.013 |
| Chao1 | 84.067 (21.572) | 87.6 (19.849) | 0.64424 | 0.42772 | 0.124 |
|  |  |  |  |  |  |
|  | **ASD FDD** | **TD FDD** | **p** | **p (adjusted)** | **Effect Size** |
| Faith-PD | 7.853 (1.395) | 8.434 (0.801) | 0.19142 | 0.43845 | 0.309 |
| Chao1 | 80.727 (19.535) | 94.8 (13.924) | 0.04192 | 0.10016 | 0.394 |
| Note. ASD: Autism Spectrum Disorder; TD: Typically Developing; Faith’s PD: Faith’s Phylogenetic Diversity. Statistical comparison was adjusted for usage of psychiatric medications, unhealthy diet, fibre intake and main nutrient intake; Effect size was calculated using rank biserial correlation. | | | | | |

| **Table S6** Summary of PERMANOVA on beta diversity indices between ASD and TD on FGID subgroups | | | | | | | | | | | | |
| --- | --- | --- | --- | --- | --- | --- | --- | --- | --- | --- | --- | --- |
| **Comparison** | | **Bray-Curtis** | | |  | **Unweighted UniFrac** | | |  | **Weighted UniFrac** | | |
| **Group 1** | **Group 2** | **F** | **p** | **p _(adjusted)_** |  | **F** | **p** | **p _(adjusted)_** |  | **F** | **p** | **p _(adjusted)_** |
| ASD FNVD | TD FNVD | 0.890 | 0.813 | 0.834 |  | 1.078 | 0.333 | 0.361 |  | 0.662 | 0.730 | 0.747 |
| ASD FAPD | TD FAPD | 1.392 | 0.046 | 0.062 |  | 1.498 | 0.071 | 0.089 |  | 0.853 | 0.488 | 0.516 |
| ASD FDD | TD FDD | 1.345 | 0.075 | 0.093 |  | 1.550 | 0.016 | **0.037** |  | 2.365 | 0.084 | 0.102 |
| Note. Permutations = 1000; adjusted for usage of psychiatric medications, unhealthy diet, fibre intake and main nutrient intake | | | | | | | | | | | | |

| **Table S7**  Comparison of the relative abundance at phylum level between ASD and TD groups | | | | | | |
| --- | --- | --- | --- | --- | --- | --- |
| **Phylum** | **Mean ± SD** | |  | **Statistics** | | |
|  | **ASD** | **TD** |  | **p** | **p(FDR)** | **Effect size** |
| Firmicutes | 63.6 (11.3) | 59.6 (13.3) |  | 0.065 | 0.108 | 0.151 |
| Bacteroidota | 14.2 (9.2) | 20.6 (10.9) |  | < .001 | **< .001** | **0.346** |
| Actinobacteriota | 18.8 (12.2) | 14 (10.4) |  | 0.004 | **0.013** | **0.235** |
| Proteobacteria | 3.1 (5) | 4.9 (8) |  | 0.012 | **0.031** | **0.203** |
| Verrucomicrobiota | 0.1 (0.4) | 0.4 (1.7) |  | 0.301 | 0.335 | 0.06 |
| Desulfobacterota | 0.1 (0.2) | 0.2 (0.4) |  | 0.002 | **0.011** | **0.222** |
| Fusobacteriota | 0.1 (0.5) | 0.2 (0.5) |  | 0.039 | 0.078 | 0.112 |
| Synergistota | 0.005 (0.03) | 0.004 (0.04) |  | 0.813 | 0.813 | 0.006 |
| Campilobacterota | 0.003 (0.03) | 0 (0) |  | 0.274 | 0.343 | 0.011 |
| Patescibacteria | 0 (0) | 0.001 (0.01) |  | 0.069 | 0.098 | 0.036 |
|  |  |  |  |  |  |  |
|  | **Mean ± SD** | |  | **Statistics** | | |
|  | **ASD FGID-** | **TD FGID-** |  | **p** | **p(FDR)** | **Effect size** |
| Firmicutes | 61.5 (11.5) | 59.8 (13.8) |  | 0.858 | 0.858 | 0.018 |
| Bacteroidota | 15.1 (9.7) | 21.1 (10.2) |  | 0.001 | **0.006** | **0.337** |
| Actinobacteriota | 19.3 (13.1) | 12.7 (9.3) |  | 0.003 | **0.013** | **0.297** |
| Proteobacteria | 3.8 (5.6) | 5.4 (8.8) |  | 0.216 | 0.36 | 0.121 |
| Verrucomicrobiota | 0.1 (0.3) | 0.4 (1.9) |  | 0.488 | 0.61 | 0.045 |
| Desulfobacterota | 0.1 (0.3) | 0.2 (0.4) |  | 0.02 | 0.065 | 0.207 |
| Fusobacteriota | 0.1 (0.6) | 0.2 (0.5) |  | 0.077 | 0.192 | 0.115 |
| Synergistota | 0.002 (0.01) | 0.005 (0.04) |  | 0.813 | 0.903 | 0.007 |
| Campilobacterota | 0.004 (0.03) | 0 (0) |  | 0.262 | 0.374 | 0.016 |
| Patescibacteria | 0 (0) | 0.001 (0.004) |  | 0.215 | 0.43 | 0.025 |
|  |  |  |  |  |  |  |
|  | **Mean ± SD** | |  | **Statistics** | | |
|  | **ASD FGID+** | **TD FGID+** |  | **p** | **p(FDR)** | **Effect size** |
| Firmicutes | 67.9 (9.9) | 59.2 (12.1) |  | 0.002 | **0.02** | **0.446** |
| Actinobacteriota | 17.7 (10.4) | 17.2 (12.1) |  | 0.629 | 0.708 | 0.073 |
| Bacteroidota | 12.4 (7.6) | 19.2 (12.6) |  | 0.031 | 0.093 | 0.319 |
| Proteobacteria | 1.5 (2.7) | 3.7 (5.4) |  | 0.008 | **0.037** | **0.39** |
| Verrucomicrobiota | 0.2 (0.6) | 0.4 (0.8) |  | 0.37 | 0.476 | 0.107 |
| Desulfobacterota | 0.055 (0.1) | 0.2 (0.4) |  | 0.066 | 0.149 | 0.233 |
| Fusobacteriota | 0.041 (0.1) | 0.2 (0.6) |  | 0.285 | 0.428 | 0.106 |
| Synergistota | 0.011 (0.06) | 0.001 (0.01) |  | 0.963 | 0.963 | 0.003 |
| Patescibacteria | 0 (0) | 0.002 (0.01) |  | 0.175 | 0.314 | 0.063 |
| Campilobacterota | 0 (0) | 0 (0) |  | - | | |
|  |  |  |  |  |  |  |
|  | **Mean ± SD** | |  | **Statistics** | | |
|  | **ASD FGID+** | **ASD FGID-** |  | **p** | **p(FDR)** | **Effect size** |
| Firmicutes | 67.9 (9.9) | 61.5 (11.5) |  | 0.007 | **0.033** | **0.347** |
| Actinobacteriota | 17.7 (10.4) | 19.3 (13.1) |  | 0.674 | 0.867 | 0.055 |
| Bacteroidota | 12.4 (7.6) | 15.1 (9.7) |  | 0.32 | 0.719 | 0.129 |
| Proteobacteria | 1.5 (2.7) | 3.8 (5.6) |  | 0.007 | 0.059 | 0.35 |
| Verrucomicrobiota | 0.2 (0.6) | 0.1 (0.3) |  | 0.273 | 0.818 | 0.094 |
| Desulfobacterota | 0.055 (0.1) | 0.1 (0.3) |  | 0.447 | 0.804 | 0.08 |
| Fusobacteriota | 0.041 (0.1) | 0.1 (0.6) |  | 0.773 | 0.87 | 0.022 |
| Synergistota | 0.011 (0.06) | 0.002 (0.01) |  | 0.968 | 0.968 | 0.002 |
| Campilobacterota | 0 (0) | 0.004 (0.03) |  | 0.501 | 0.752 | 0.016 |
| Patescibacteria | 0 (0) | 0 (0) |  | - | | |
|  |  |  |  |  |  |  |
|  | **Mean ± SD** | |  | **Statistics** | | |
|  | **TD FGID+** | **TD FGID-** |  | **p** | **p(FDR)** | **Effect size** |
| Firmicutes | 59.2 (12.1) | 59.8 (13.8) |  | 0.74 | 0.952 | 0.041 |
| Bacteroidota | 19.2 (12.6) | 21.1 (10.2) |  | 0.379 | 0.683 | 0.107 |
| Actinobacteriota | 17.2 (12.1) | 12.7 (9.3) |  | 0.07 | 0.628 | 0.22 |
| Proteobacteria | 3.7 (5.4) | 5.4 (8.8) |  | 0.102 | 0.458 | 0.108 |
| Verrucomicrobiota | 0.4 (0.8) | 0.4 (1.9) |  | 0.376 | 0.845 | 0.147 |
| Desulfobacterota | 0.2 (0.4) | 0.2 (0.4) |  | 0.598 | 0.898 | 0.06 |
| Fusobacteriota | 0.2 (0.6) | 0.2 (0.5) |  | 0.898 | 0.898 | 0.012 |
| Synergistota | 0.001 (0.01) | 0.005 (0.04) |  | 0.881 | 0.991 | 0.005 |
| Patescibacteria | 0.002 (0.01) | 0.001 (0.004) |  | 0.331 | 0.994 | 0.038 |
| Campilobacterota | 0 (0) | 0 (0) |  | - | | |
| Note. ASD: Autism Spectrum Disorder; TD: Typically Developing; FGID: Functional Gastrointestinal Disorder. P-values were corrected for false discovery rate using the Benjamini-Hochberg method. Effect sizes were calculated with rank biserial correlation. Phyla are presented in descending order of relative abundance. | | | | | | |

| **Table S8** | | | | | | |
| --- | --- | --- | --- | --- | --- | --- |
| Comparison of the relative abundance at genus level between ASD and TD | | | | | | |
| Genera | Mean ± SD | |  | Statistics | | |
|  | ASD | TD |  | p | p _FDR_ | Effect size |
| Bifidobacterium | 18.4 (12) | 13.3 (10.3) |  | 0.002 | **0.008** | **0.254** |
| Bacteroides | 12.3 (9.01) | 16.5 (10.3) |  | 0.002 | **0.013** | **0.247** |
| Blautia | 10.2 (5.43) | 8.08 (4.07) |  | 0.007 | **0.019** | **0.22** |
| Parabacteroides | 0.741 (1.17) | 1.21 (1.39) |  | 0.002 | **0.01** | **0.243** |
| Enterobacter | 0.506 (2.23) | 0.392 (0.949) |  | 0.031 | **0.037** | **0.128** |
| Alistipes | 0.424 (0.729) | 0.96 (1.3) |  | 0.001 | **0.01** | **0.254** |
| Phascolarctobacterium | 0.343 (0.784) | 0.714 (0.956) |  | 0.001 | **0.019** | **0.238** |
| Collinsella | 0.33 (0.671) | 0.478 (0.604) |  | 0.008 | **0.017** | **0.188** |
| Dorea | 0.19 (0.532) | 0.042 (0.202) |  | 0.015 | **0.022** | **0.102** |
| Bilophila | 0.0951 (0.222) | 0.172 (0.295) |  | 0.012 | **0.019** | **0.173** |
| Fusobacterium | 0.0943 (0.459) | 0.215 (0.533) |  | 0.038 | **0.042** | **0.112** |
| Sutterella | 0.0622 (0.229) | 0.187 (0.436) |  | 0.007 | **0.017** | **0.156** |
| Ruminococcaceae (family) | 0.0219 (0.0757) | 0.0123 (0.0838) |  | 0.03 | **0.038** | **0.089** |
| Allisonella | 0.00791 (0.0622) | 0.033 (0.124) |  | 0.027 | **0.037** | **0.076** |
| RF39 | 0.00612 (0.0587) | 0.0265 (0.133) |  | 0.039 | **0.041** | **0.06** |
| Weissella | 0.00121 (0.00858) | 0.102 (0.424) |  | 0.009 | **0.017** | **0.096** |
| Eggerthellaceae uncultured | 0 (0) | 0.0308 (0.127) |  | 0.006 | **0.019** | **0.08** |
| Coprobacter | 0 (0) | 0.00575 (0.0296) |  | 0.041 | **0.041** | **0.045** |
| [Eubacterium] ruminantium group | 0 (0) | 0.0589 (0.248) |  | 0.009 | **0.016** | **0.071** |
| Note. ASD: Autism Spectrum Disorder; TD: Typically Developing. P-values were corrected for false discovery rate using the Benjamini-Hochberg method. Effect sizes were calculated with rank biserial correlation. Genera are presented in descending order of relative abundance. | | | | | | |

| **Table S9** | | | | | | |
| --- | --- | --- | --- | --- | --- | --- |
| Comparison of the relative abundance at genus level between TD FGID+ and TD FGID- | | | | | | |
|  | Mean ± SD | |  | Statistics | | |
|  | TD FGID+ | TD FGID- |  | p | p _FDR_ | Effect size |
| [Eubacterium] hallii group | 1.813 (1.51) | 1.099 (1.10) |  | 0.023 | 0.115 | 0.271 |
| Parabacteroides | 1.002 (1.55) | 1.291 (1.32) |  | 0.048 | **0.048** | **0.239** |
| Roseburia | 0.652 (0.99) | 1.549 (1.79) |  | 0.029 | **0.041** | **0.252** |
| [Eubacterium] eligens group | 0.013 (0.05) | 0.269 (0.65) |  | 0.032 | **0.04** | **0.174** |
| Barnesiella | 0.152 (0.42) | 0.041 (0.26) |  | 0.028 | **0.047** | **0.119** |
| Colidextribacter | 0.029 (0.12) | 0.087 (0.18) |  | 0.023 | 0.077 | 0.207 |
| [Eubacterium] fissicatena group | 0.050 (0.20) | 0 (0) |  | 0.026 | 0.065 | 0.063 |
| Christensenellaceae uncultured | 0.026 (0.11) | 0.002 (0.01) |  | 0.035 | **0.039** | **0.1** |
| Anaerofustis | 0.013 (0.03) | 0.002 (0.01) |  | 0.008 | 0.08 | 0.152 |
| UCG-009 | 0.006 (0.02) | 0 (0) |  | 0.026 | 0.052 | 0.063 |
| Note. TD: Typically Developing; FGID: Functional Gastrointestinal Disorder. P-values corrected for False Discovery Rate using the Benjamini-Hochberg method. Effect size was calculated with rank biserial correlation. Genera are presented in descending order of relative abundance. | | | | | | |

| **Table S10** Correlation matrix between alpha diversity indices, *Firmicutes: Bacteroidetes* ratio and clinical characteristics of ASD | | | | |
| --- | --- | --- | --- | --- |
|  |  | **AQ-10** | **SCAS-P** | **SDQ** |
| Faith-PD | Spearman's rho | -0.212 | -0.002 | -0.143 |
|  | p-value | **0.002** | 0.978 | **0.045** |
| Chao1 | Spearman's rho | -0.238 | -0.068 | -0.197 |
|  | p-value | **0.001** | 0.335 | **0.005** |
| F:B ratio | Spearman's rho | 0.146 | 0.020 | 0.230 |
|  | p-value | **0.038** | 0.779 | **0.001** |
| Note. AQ-10: Autism Spectrum Quotient 10 items; F:B ratio: *Firmicutes: Bacteroidetes* ratio; SCAS-P: Spence Children's Anxiety Scale - Parent version; SDQ: Strengths and Difficulties Questionnaire - Parent version | | | | |


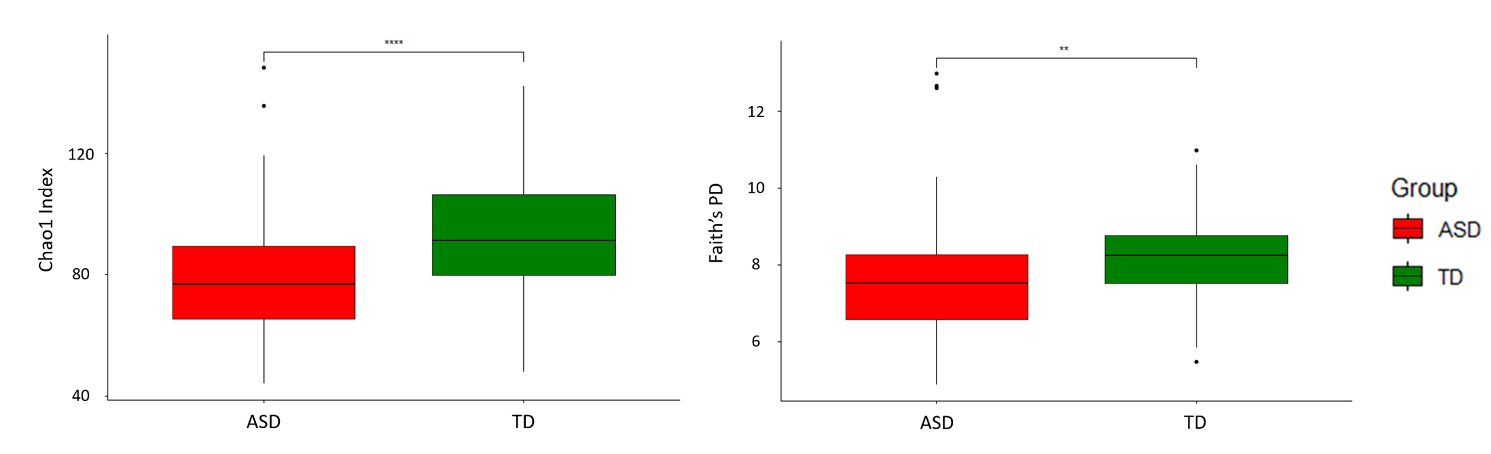


Figure S1. Comparison of alpha diversity (the Chao1 Index and the Faith’s Phylogenetic Diversity) between ASD and TD; * p < .05, ** p < .01, *** p < .001, **** p < .0001.


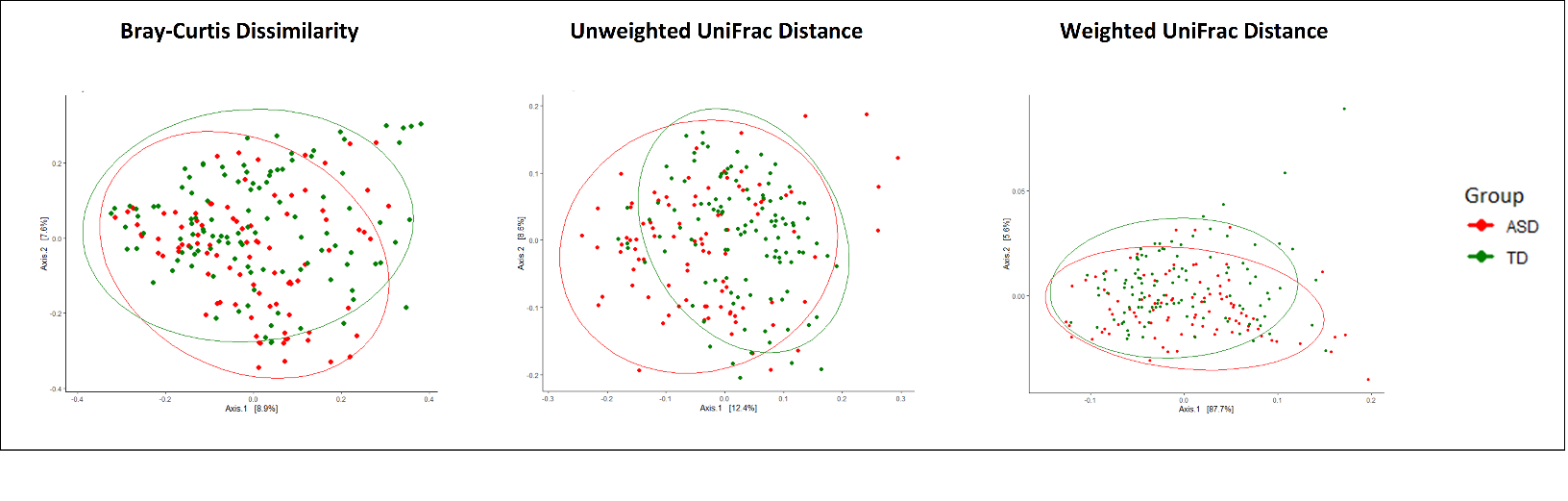


Figure S2. Principal coordinates analysis based on the Bray-Curtis dissimilarity, unweighted and weighted UniFrac distances between ASD and TD.


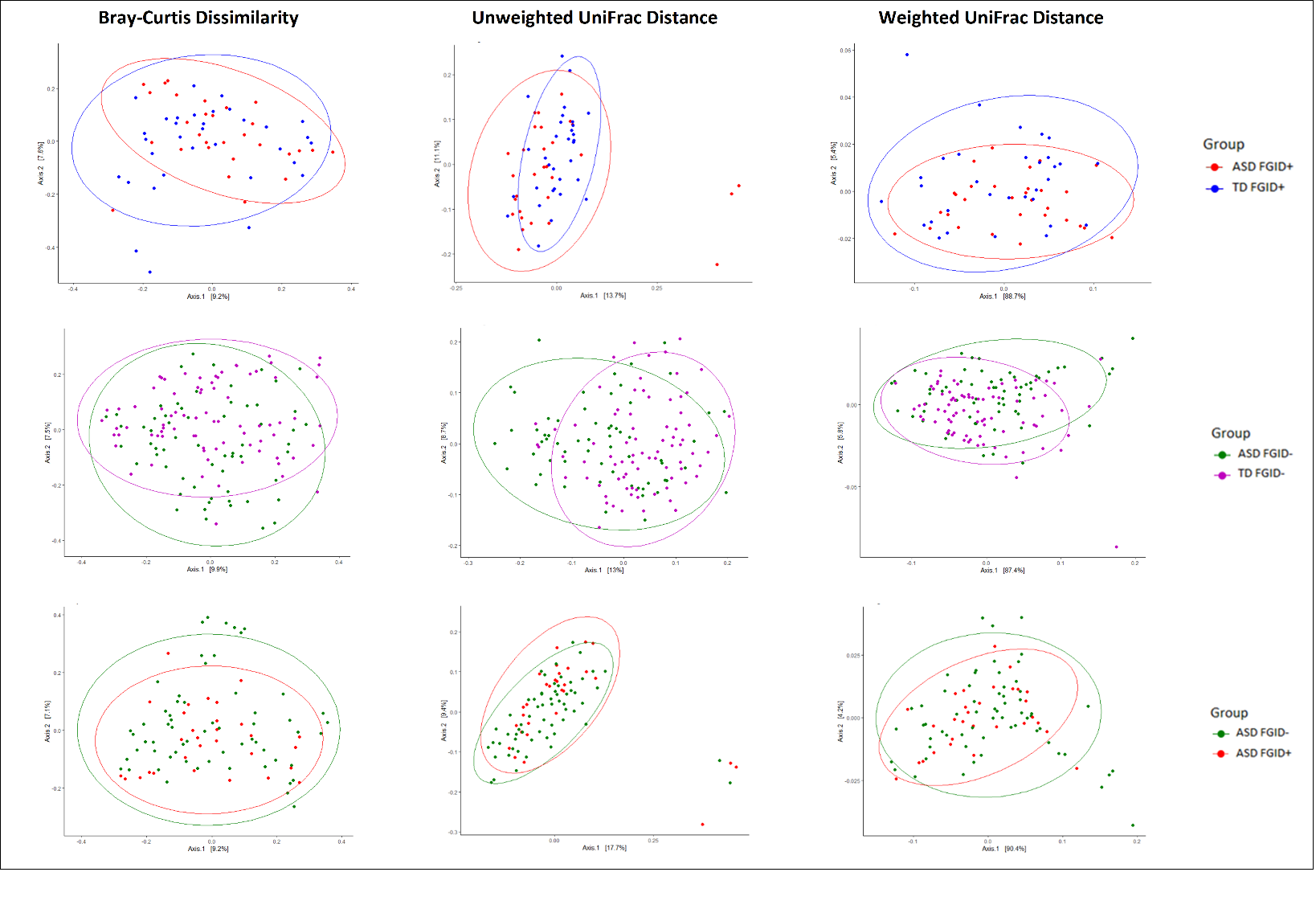


Figure S3. Principal coordinates analysis based on the Bray-Curtis dissimilarity, unweighted and weighted UniFrac distances between 1. ASD FGID+ vs. TD FGID+ (top row), 2 ASD FGID- vs. TD FGID- (middle row), and 3. ASD FGID+ vs. ASD FGID- (bottom row).


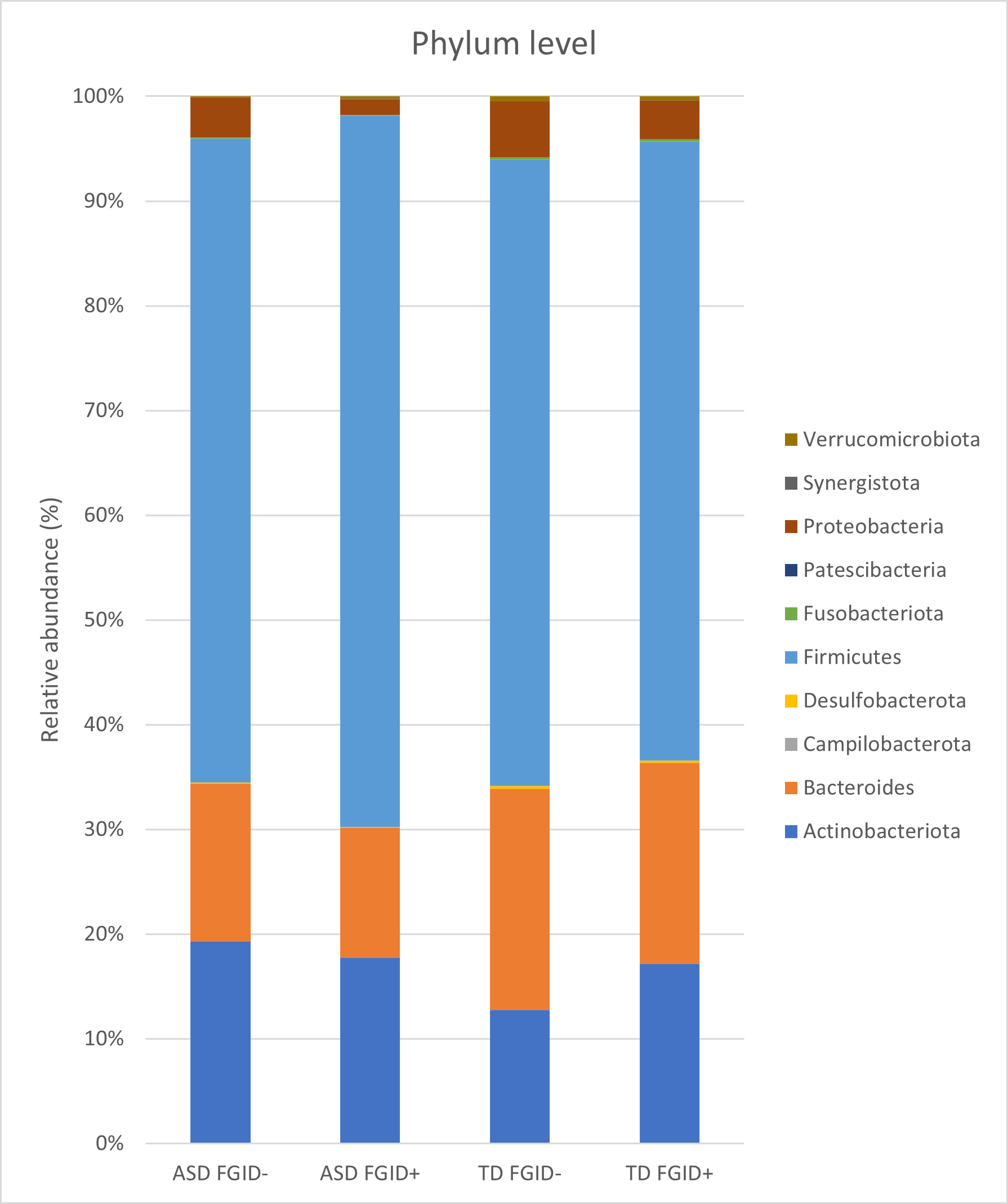


Figure S4. Comparison of gut microbiota relative abundance at the phylum level between ASD FGID-, ASD FGID+, TD FGID-, and TD FGID+.

Dietary Questionnaire by the Department of Health of the Hong Kong SAR government
Department of Health. Child Health Survey 2005-2006 https://www.chp.gov.hk/files/pdf/chs_eng.pdf. (2010).

In the past 7 days and on average,

| 1. How many cup(s) of milk does your child drink | Never^1^ | <1 cup^2^ | | 1 cup^3^ | | | 2cups^4^ | >3cups^5^ | |
| --- | --- | --- | --- | --- | --- | --- | --- | --- | --- |
| 2. How many cup(s) of fruit juice does your child drink | Never^1^ | <1 cup^2^ | | 1 cup^3^ | | | 2cups^4^ | >3cups^5^ | |
| 3. How many cup(s) of soft drinks does your child drink | Never^1^ | <1 cup^2^ | | 1 cup^3^ | | | 2cups^4^ | >3cups^5^ | |
| Note: 1 cup = 250ml ~8oz | | | | | | | | | |
| 4. How many portion of fruit(s) does your child consume | Never or very infrequent^1^ | <1 cup^2^ | | 1 cup^3^ | | | 2cups^4^ | >3cups^5^ | |
| Note: 1 portion = 1 orange/apple/pear or 1 large slides of watermelon/melon or 2 plums/ tangerine or ½ cup of grapes/cherry | | | | | | | | | |
| 5. How much vegetable does your child consume every meal? | Never or very infrequent^1^ | <1/2 bowl per meal^2^ | 1/2 bowl per meal^3^ | 3/4 bowl per meal^4^ | | 1 bowl per meal^5^ | | >1 bowl per meal^6^ | |
| 6. How much cereals does your child consume every meal? (Including cereal breakfast, bread, rice, noodle, pasta and etc.)? (3 pieces of bread=1 bowl of rice) | Never or very infrequent^1^ | <1/2 bowl per meal^2^ | 1/2 bowl per meal^3^ | 3/4 bowl per meal^4^ | | 1 bowl per meal^5^ | | >1 bowl per meal^6^ | |
| 7. What is the average amount of the following items would your child consume? | | | | | | | | | |
| 1. Meat | Never^1^ | | | At least 100g per meal^2^ | | | | | |
| 1. Fish | Never^1^ | | | At least 100g per meal^2^ | | | | | |
| 1. Beans | Never^1^ | | | At least 1 bowl per week^2^ | | | | | |
| 1. Egg | Never^1^ | | | At least 1 per week^2^ | | | | | |
| 8. How many times per week does your child consume meals in fast-food shop? | Never^1^ | 1^2^ | 2^3^ | 3^4^ | 4^5^ | | 5^6^ | 6^7^ | 7^8^ |
| 9. How many times does your child consume snacks (e.g. chips, candy and biscuit) in a week? | Never^1^ | 1-3times per week^2^ | | 4-6 times per week^3^ | | | At least once per day^4^ | | |
| 10. How frequent does your child consume deep-fried food in the regular meals? | ≤ 2 times per week^1^ | 3-5 times per week^2^ | | >5 times per week^3^ | | | Never^4^ | | |
| 11. When you feed your child with meat which contains fatty meat, would you | Remove all the fatty meat before letting the child consume^1^ | Remove part of the fatty meat before letting the child consume^2^ | | Let the child consume the fatty meat^3^ | | | Never would let the child consume any meat with fatty meat^4^ | | |

Scoring algorithm for the dietary questionnaire:

//Consumption of main nutrients//
recode Question 5 6 (1=0) (2=0) (3=0) (4=0) (5=1) (6=1) into Dn05x Dn06x.

recode Question 4 (1=0) (2=0) (3=1) (4=1) (5=1) into Dn04x.

recode Question 7a 7b 7c 7d (1=0) (2=1) into Dn07ax Dn07bx Dn07cx Dn07dx.

recode Question 1 (1=0) (2=0) (3=0) (4=1) (5=1) into Dn01x.

variable labels
Dn01x "Consumption of milk for at least 2 cups each day"
Dn04x "Consumption of fruits for at least 1 unit per day".

Dn05x "Consumption of vegetables for at least 1 bowl per meal"
Dn06x "Consumption of cereals for at least 1 bowl per meal".

Dn07ax "Consumption of meat in the 7 days preceding the survey" Dn07bx "Consumption of fish in the 7 days preceding the survey".

Dn07cx "Consumption of beans in the 7 days preceding the survey" Dn07dx "Consumption of eggs in the 7 days preceding the survey".

//Consumption of food rich in sugar, salt, fat//
recode Question 3 (1=0) (2=0) (3=1) (4=1) (5=1) into Dn03x.
recode Question 8 (1=0) (2=0) (3=1) (4=1) (5=1) (6=1) (7=1) (8=1) into Dn08x.
recode Question 9 (1=0) (2=0) (3=0) (4=1) into Dn09x.
recode Question 10 (1=0) (2=1) (3=1) (4=0) into Dn10x.
recode Question 11 (1=0) (2=0) (3=1) (4=0) into Dn11x.
variable labels
Dn03x "Consumption of soft drinks for at least 1 cup each day"
Dn08x "Consumption of fast food for at least 2 times per week".

Dn09cx "Consumption of junk food for at least once a day"
Dn10x "Consumption of fried food in main meals for at least 3 times per week".
Dn11x "Fatty part of meat not removed by parents".

//Composite Scores calculation//

1. Fiber
   compute Fiber=(Dn04x+Dn05x).
2. Main Nutrients
   compute Main Nutrients =(Dn01x+Dn04x+Dn05x+Dn06x+Dn07ax+Dn07bx+Dn07cx+Dn07dx).
3. Unhealthy Food
   compute Unhealthy Food =(Dn03x+Dn08x+Dn09x+Dn10x+Dn11x).
